# New possibilities for chromoblastomycosis and phaeohyphomycosis treatment: identification of two compounds from the MMV Pathogen Box^®^ that present synergism with itraconazole

**DOI:** 10.1590/0074-02760220089

**Published:** 2022-09-12

**Authors:** Rowena Alves Coelho, Gabriela Machado Alves, Maria Helena Galdino Figueiredo-Carvalho, Fernando Almeida-Silva, Gabriela Rodrigues de Souza, Maria Cristina da Silva Lourenço, Fábio Brito-Santos, Ana Claudia Fernandes Amaral, Rodrigo Almeida-Paes

**Affiliations:** 1 Fundação Oswaldo Cruz-Fiocruz, Instituto Nacional de Infectologia Evandro Chagas, Laboratório de Micologia, Rio de Janeiro, RJ, Brasil; 2 Fundação Oswaldo Cruz-Fiocruz, Instituto Nacional de Infectologia Evandro Chagas, Plataforma de Bioensaios RPT 11B, Rio de Janeiro, RJ, Brasil; 3 Hospital Universitário Pedro Ernesto, Rio de Janeiro, RJ, Brasil; 4 Fundação Oswaldo Cruz-Fiocruz, Farmanguinhos, Laboratório de Produtos Naturais e Derivados, Rio de Janeiro, RJ, Brasil

**Keywords:** Exophiala, Fonsecaea, synergism, drug repurposing, itraconazole, Pathogen Box

## Abstract

**BACKGROUND:**

Black fungi of the Herpotrichiellaceae family are agents of chromoblastomycosis and phaeohyphomycosis. There are few therapeutic options for these infections and it is common to associate antifungal drugs in their treatment.

**OBJECTIVES:**

To investigate the Medicines for Malaria Venture (MMV) Pathogen Box^®^ for possible compounds presenting synergism with antifungal drugs used to treat black fungal infections.

**METHODS:**

An initial screening of the Pathogen Box^®^ compounds was performed in combination with itraconazole or terbinafine at sub-inhibitory concentrations against *Fonsecaea pedrosoi*. Hits were further tested against eight Herpotrichiellaceae using the checkerboard method.

**FINDINGS:**

No synergism was observed with terbinafine. MMV687273 (SQ109) and MMV688415 showed synergism with itraconazole against *F. pedrosoi*. Synergism of these compounds was confirmed with some black fungi by the checkerboard method. SQ109 and itraconazole presented synergism for *Exophiala dermatitidis*, *F. pedrosoi*, *F. monophora* and *F. nubica*, with fungicidal activity for *F. pedrosoi* and *F. monophora*. MMV688415 presented synergism with itraconazole only for *F. pedrosoi*, with fungicidal activity. The synergic compounds had high selectivity index values when combined with itraconazole.

**MAIN CONCLUSIONS:**

These compounds in combination, particularly SQ109, are promising candidates to treat *Fonsecaea* spp. and *E. dermatitidis* infections, which account for most cases of chromoblastomycosis and phaeohyphomycosis.

Many species of melanised filamentous fungi and black yeasts that belong to the Herpotrichiellaceae family are agents involved in subcutaneous, systemic or disseminated infections, known as chromoblastomycosis (CBM) or phaeohyphomycosis (PHM).^1^ ^,^ ^2^ ^,^ ^3^ ^,^ ^4^ Some species of this family also cause eumycotic mycetoma.^5^

CBM is an implantation neglected disease, caused by melanised fungi widely found in nature which infect mainly agricultural workers after transcutaneous inoculation,^2^ ^,^ ^6^ ^,^ ^7^ ^,^ ^8^ thus constituting an occupational disease.^9^ This mycosis is prevalent in tropical and subtropical regions of the world, especially in Madagascar, India, China, Japan, Australia, South Africa, Mexico, Cuba, Dominican Republic, Venezuela, and Brazil.^2^ ^,^ ^8^ In Brazil, cases are reported in the Amazon Region, the main endemic area, as well as in the states of Minas Gerais, Goiás, Rio Grande do Sul, Paraná, São Paulo, Maranhão, and Rio de Janeiro.^10^ ^-^ ^16^ Species of the genus *Fonsecaea* are the main agents of this infection.^8^

PHM is also caused by melanised fungi that infect humans through traumatic inoculation. However, it comprises a group of mycotic infections that contain dematiaceous yeast like cells, pseudohyphae-like elements, hyphae, or any combination of these forms in the tissue.^17^ ^,^ ^18^ ^,^ ^19^ The terms superficial, cutaneous and corneal, subcutaneous and systemic PHM are proposed for the main categories of PHM.^20^ Invasive PHM is mainly caused by *Bipolaris* spp. and *Exophiala* spp., and is usually severe in both immunocompetent and immunocompromised patients.^21^ ^,^ ^22^ The most frequently reported agents of subcutaneous PHM are *Exophiala jeanselmei*, *Exophiala dermatitidis* and *Phialophora verrucosa*.^23^

The treatment of infections caused by black fungi has always been considered a challenge because there are few therapeutic options and physical treatment methods are usually used together with oral antifungal therapy.^2^ ^,^ ^24^ ^,^ ^25^ In addition, it is common to associate more than one antifungal drug in the treatment of CBM, in addition to the largely used itraconazole and terbinafine.^26^ 5-Fluorocytosine (5FC) and amphotericin B,^27^ itraconazole and 5FC,^28^ and terbinafine and amphotericin^29^ are examples of antifungal combinations used in CBM treatment. Other combinations with second-generation triazoles such as terbinafine and voriconazole are successfully used, however the expensiveness of these medications makes their use impractical in most cases.^30^ ^,^ ^31^ ^,^ ^32^

Recently, some studies have been developed in search of new active molecules against agents of several mycoses, showing the antifungal activity of some compounds previously used for other medical purposes, the so-called drug repositioning.^33^ ^-^ ^38^ However, in the context of infections caused by dematiaceous fungi, the development of new diagnostics, drugs and other control tools are neglected research topics. To contribute with this field, our group identified eight compounds in the Medicines for Malaria Venture (MMV) Pathogen Box^®^ with antifungal activity against members of the Herpotrichiellaceae family, being auranofin and iodoquinol the most promising drugs for future therapies.^37^ In order to improve this knowledge, we further investigated this substance library to identify possible synergisms of their 400 compounds with itraconazole or terbinafine, the main antifungal drugs used to treat black fungi infections.

## MATERIALS AND METHODS

*Fungal strains and growth conditions* - The eight reference strains used in the study were obtained from the Collection of Pathogenic Fungi (CFP), National Institute of Infectious Diseases Evandro Chagas, Oswaldo Cruz Foundation, Rio de Janeiro, Brazil. *Fonsecaea pedrosoi* CFP 00791, the most common CBM agent, was used throughout the study. In addition, *Cladophialophora carrionii* CFP 00910, *P. verrucosa* CFP 00937, *Fonsecaea monophora* CFP 00911, *Fonsecaea nubica* CFP 00912, *Rhinocladiela similis* CFP 00790, *Exophiala heteromorpha* CFP 01088, and *E. dermatitidis* CFP 01087 were used for synergism assays. These strains comprise several agents of CBM and/or PHM. Additionaly, six clinical strains, obtained from CBM cases, were studied to confirm synergism of the most promising drug combination. These strains are as follows: *F. nubica* 34242, *F. monophora* 34904, *F. monophora* 36134, *F. monophora* 36831, *F. monophora* 41080, and *F. pedrosoi* 38714. Strains were maintained on potato dextrose agar (PDA) (Sigma Chemical Corporation, St. Louis, MO, USA) and seven-day-old cultures incubated at 30ºC were used in the assays. Minimum inhibitory concentration (MIC) values of *F. pedrosoi* CFP 00791 for itraconazole and terbinafine were previously described,^37^ which were 0.34 and 0.73 µM, respectively.

*Pathogen Box* ^®^ *compound library* - The Pathogen Box^®^ was kindly provided by MMV (Geneva, Switzerland). It holds 400 different compounds with proven activity against agents of neglected diseases, such as malaria, tuberculosis, and Chagas disease, and with cytotoxicity levels considered adequate for an initial drug discovery plan.^39^ The library was supplied in 96-well microtiter plates containing 10 µL/well of 10 mM compound solutions in dimethylsulfoxide (DMSO). These compounds were diluted according to the manufacturer’s initial protocol.

*Screening for synergistic interaction* - A screening of Pathogen Box^®^ compounds was performed in combination with itraconazole or terbinafine (both from Sigma-Aldrich, St Louis, USA) at sub-inhibitory concentrations (0.085 µM and 0.182 µM, respectively). In summary, for preparation of the fungal inoculum, *F. pedrosoi* CFP 00791 was grown as described above and the conidia were suspended in sterile distilled water supplemented with 0.1% Tween 20 (Sigma Chemical Corporation), with the suspension turbidity adjusted to the 0.5 McFarland scale. This suspension was then diluted 1:10 to obtain a final working inoculum of 2 - 5 × 10^5^ CFU/mL.^40^ Then, 100 µL of the fungal inoculum was added to each well containing the compounds, the plates were incubated at 35ºC for 72-96 hours and visual reading was performed observing the wells with 100% inhibition. The eight compounds that, when tested alone, presented antifungal activity against *F. pedrosoi* in our previous study^37^ were excluded from further analyses.

*Confirmation of synergistic effects* - Synergistic activity of the compounds identified in the screening was confirmed by the checkerboard method.^41^ In this test, two drugs are loaded into a single 96-well plate, so that in each well there are different concentrations of the compound-antifungal combination. Compound-antifungal dilutions were prepared following the methodology proposed by the EUCAST, starting from a 100-fold concentrated stock compound-antifungal solution.^40^ Final concentrations of the antifungal drug and MMV compounds corresponded to 0.015 - 8 µM and 0.25 - 16 µM, respectively. Fungal inocula and incubation conditions were the same described for the screening. MIC were defined as the lowest drug concentration that was able to completely inhibit fungal growth. The drug interaction was classified according to the fractional inhibitory concentration index (FICI). The FICI was obtained by the formula: FICI ═ (A/MIC (a)) + (B/MIC (b)), where: A = MIC of the drug (a) in combination; MIC (a) = MIC of drug (a) alone; B = MIC of the drug (b) in combination; MIC (b) = MIC of drug (b) alone.^42^ The type of interaction between the tested compounds was classified as synergism if FICI ≤ 0.5; as indifferent if *>* 0.5 and *<* 4; and as antagonism if FICI *≥* 4.^41^ ^,^ ^42^ This experiment was performed initially with the reference strains to confirm the synergistic effects of the combinations hits found in the screening with *F. pedrosoi* with more agents of black mould human mycoses, and later with strains isolated from CBM cases, to discard possible strain-specific effects in the most promising drug combination.

*Fungicidal activity* - The minimal fungicidal concentration (MFC) was determined by transferring an aliquot of 5 µL of each well without fungal growth of the microdilution plates used for FICI determination (FFCI), similar as described above, on Sabouraud 2% glucose agar (Sigma Chemical Corporation). The MFC was determined as the lowest drug concentration without fungal growth on the Sabouraud agar after five days of incubation at 35ºC. Compounds that presented a MFC value lower than two times the MIC were considered fungicidal.^43^ The fractional fungicidal concentration index (FFCI) was calculated as FFCI ═ (A/MFC (a)) + (B/MFC (b)), where: A = MFC of the drug (a) in combination; MFC (a) = MFC of drug (a) alone; B = MFC of the drug (b) in combination; MFC (b) = MFC of drug (b) alone.^44^

*Cytotoxicity evaluation* - VERO cells (ATCC CCL-81, a kidney tissue derived from a normal, adult African green monkey), were cultured in medium 199 with Earle’s salts complemented with 100 U/mL penicillin-100 µg/mL streptomycin (Cultilab LTDA, Brazil) and 10% foetal bovine serum (FBS, Cultilab LTDA, Brazil) in a 37ºC incubator with 5% CO_2_. Cells were sub-cultured in 25 or 75 cm^2^ culture flasks once a week and the culture medium was changed at the same rate. The cells used in the experiments were from passages 10 through 29.^45^ Cytotoxicity assays to evaluate cell viability percentage were performed in 96-well plates containing 5 × 10^4^ cells/well that were exposed to treatment solutions for 24 h at 37ºC in an incubator with 5% CO_2_. MTT formazan powder (Sigma-Aldrich^®^, USA) was used and a 10% Tween 80 solution was the positive control. Results were read at 492 nm in a Thermo Scientific^®^ Multiskan microplate spectrophotometer reader and expressed as % of cell viability in the culture medium after addition of a 10% Tween 80 solution.^46^

Initially, cells were treated with a range of itraconazole and SQ109 concentrations (100-0.001 µM) and SQ109 combined to itraconazole (100:50 to 0.001:0.0005), respectively to find the No Observed Effect Concentrations (NOEC) and CC_50_ (concentration that inhibits 50% of growth). Controls received only the culture medium without or with DMSO 1%. The selectivity index (SI) was calculated using the formula: SI = CC_50_ (µM) / MIC (µM). The higher the ratio obtained, the more selective is the substance against the pathogen.

## RESULTS

**Fig. 1:**
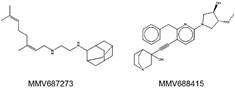
chemical structure of the Medicines for Malaria Venture (MMV) compounds with synergism with itraconazole against agents of chromoblastomycosis and phaeohyphomycosis. (A) MMV887273, also known as SQ109; (B) MMV688415.

*The MMV Pathogen Box* ^®^ *has two compounds presenting synergism with itraconazole* - The screening of the MMV Pathogen Box^®^ compounds in combination with terbinafine found no synergistic compound against *F. pedrosoi* CFP 00791. However, two compounds showed synergism with itraconazole: MMV687273, also known as SQ109, and MMV688415 (Fig. 1). Their original applications, as described by MMV, are as follows: anti-tuberculosis and anti-kinetoplastids, respectively.

*MMV687273 has a broader synergistic effect than MMV688415* - The FICI of eight Herpotrichiellaceae species were determined to confirm and check the extent of the synergism found during the screening with the *F. pedrosoi* CFP 00791 strain. Table I presents detailed information about the combination of the two compounds of the MMV Pathogen Box^®^ and itraconazole against the black fungi studied herein. A synergistic interaction was found in the combination of SQ109 and itraconazole for four strains: *E. dermatitidis* CFP 01087, *F. pedrosoi* CFP 00791, *F. monophora* CFP 00911 and *F. nubica* CFP 00912. In the combination of MMV688415 with itraconazole, a synergistic interaction was found only for the strain *F. pedrosoi* CFP 00791. To confirm the synergism found between SQ109 and itraconazole, other clinical strains of *Fonsecaea* spp. (four *F. monophora*, one *F. nubica* and one *F. pedrosoi*). Of these, four strains (66.66%) showed synergism, corroborating our initial findings (Table II).

**TABLE I:** Fractional inhibitory concentration index (FICI) for MMV687273 (SQ109) and MMV688415 in combination with itraconazole (ITZ) against eight Herpotrichiellaceae species of clinical interest

Strains	MIC (µM)			FICI SQ109/ITZ	MIC (µM)			FICI 688415/ITZ
	SQ109	ITZ	SQ109/ITZ		688415	ITZ	688415/ITZ	
*Cladophialophora carrionii*	2.0	0.25	1.0/0.125	1	≥ 32	0.25	0,25/0,25	1.007
*Phialophora verrucosa*	4.0	1.0	0.015/4.0	1.015	≥ 32	1.0	1.0/0.5	0.56
*Exophiala dermatitidis*	4.0	1.0	0.5/0.125	0.25*	≥ 32	1.0	0.25/1.0	1.01
*Exophiala heteromorpha*	16	0.50	16/0.015	1.03	≥ 32	0.50	0.25/0.50	1.01
*Fonsecaea pedrosoi*	4.0	0.50	0.50/0.0625	0.25	≥ 32	0.50	2.0/0.125	0.37
*Fonsecaea monophora*	4.0	0.50	0.25/0.125	0.31	≥ 32	0.25	0.25/0.25	1.01
*Fonsecaea nubica*	4.0	0.25	1.0/0.0625	0.50	≥ 32	0.50	0.25/0.50	1.01
*Rhinocladiella similis*	8.0	0.50	4.0/0.125	0.75	≥ 32	0.50	0.25/0.50	1.01

^*^Bold FICI values indicate synergistic interactions. MIC: minimum inhibitory concentration.

**TABLE II:** Fractional inhibitory concentration index (FICI) for MMV687273 (SQ109) in combination with itraconazole (ITZ) against six *Fonsecaea* spp. clinical strains

Strains	MIC (µM)			FICI SQ109/ITZ
	SQ109	ITZ	SQ109/ITZ	
34242 *Fonsecaea nubica*	4.0	0.50	1.0/0.125	0.50*
34904 *Fonsecaea monophora*	4.0	0.25	1.0/0.125	0.75
36134 *Fonsecaea monophora*	4.0	0.25	1.0/0.03125	0.37
36831 *Fonsecaea monophora*	4.0	0.25	1.0/0.125	0.75
38714 *Fonsecaea pedrosoi*	4.0	0.25	1.0/0.0625	0.50
41080 *Fonsecaea monophora*	4.0	0.25	1.0/0.0625	0.50

MIC: minimum inhibitory concentration.

*Some drug combinations have fungicidal activity* - Table III depicts the FFCI of the studied combinations against the eight fungal species. SQ109, in combination with itraconazole, was fungicidal for *F. pedrosoi* and *F. monophora* strains, while MMV688415 in combination with itraconazole was fungicidal only for *F. pedrosoi*. When analysing these compounds separately, we observed that itraconazole was fungistatic for all strains while SQ109 was fungicidal for *C. carrionii*, *P. verrucosa*, *F. pedrosoi*, *F. monophora* and *R. similis* strains*.* MMV688415 showed growth at all concentrations tested so it was not possible to calculate its exact MFC (> 16 µM).

**TABLE III:** Fractional fungicidal concentration index (FFCI) for MMV687273 (SQ109) and MMV688415 in combination with itraconazole (ITZ) against eight Herpotrichiellaceae species of clinical interest

Strains	MFC (µM)			FFCI SQ109/ITZ	MFC (µM)			FFCI 688415/ITZ
	SQ109	ITZ	SQ109/ITZ		688415	ITZ	688415/ITZ	
*Cladophialophora carrionii*	2.0	1.0	1.0/0.125	0.62	≥ 32	2.0	8.0/1.0	0.75
*Phialophora verrucosa*	8.0	4.0	16/4.0	3	≥ 32	1.0	1.0/2.0	2.015
*Exophiala heteromorpha*	16	8.0	16/0.125	1.015	≥ 32	8.0	16/1.0	0.62
*Exophiala jeanselmei*	≥32	8.0	16/4.0	1	≥ 32	8.0	0.25/4.0	0.507
*Fonsecaea pedrosoi*	4.0	2.0	0.0625/0.50	0.26*	≥ 32	2.0	2.0/0.125	0.25
*Fonsecaea monophora*	4.0	2.0	0.25/0.125	0.12	≥ 32	2.0	4.0/2.0	1.125
*Fonsecaea nubica*	16	2.0	1.0/2.0	1.062	≥ 32	4.0	16/1.0	0.75
*Rhinocladiella similis*	8.0	8.0	4.0/8.0	1.50	≥ 32	4.0	16/2.0	1

^*^Bold FFCI values indicate synergistic interactions. MFC: minimal fungicidal concentration.

**Fig. 2:**
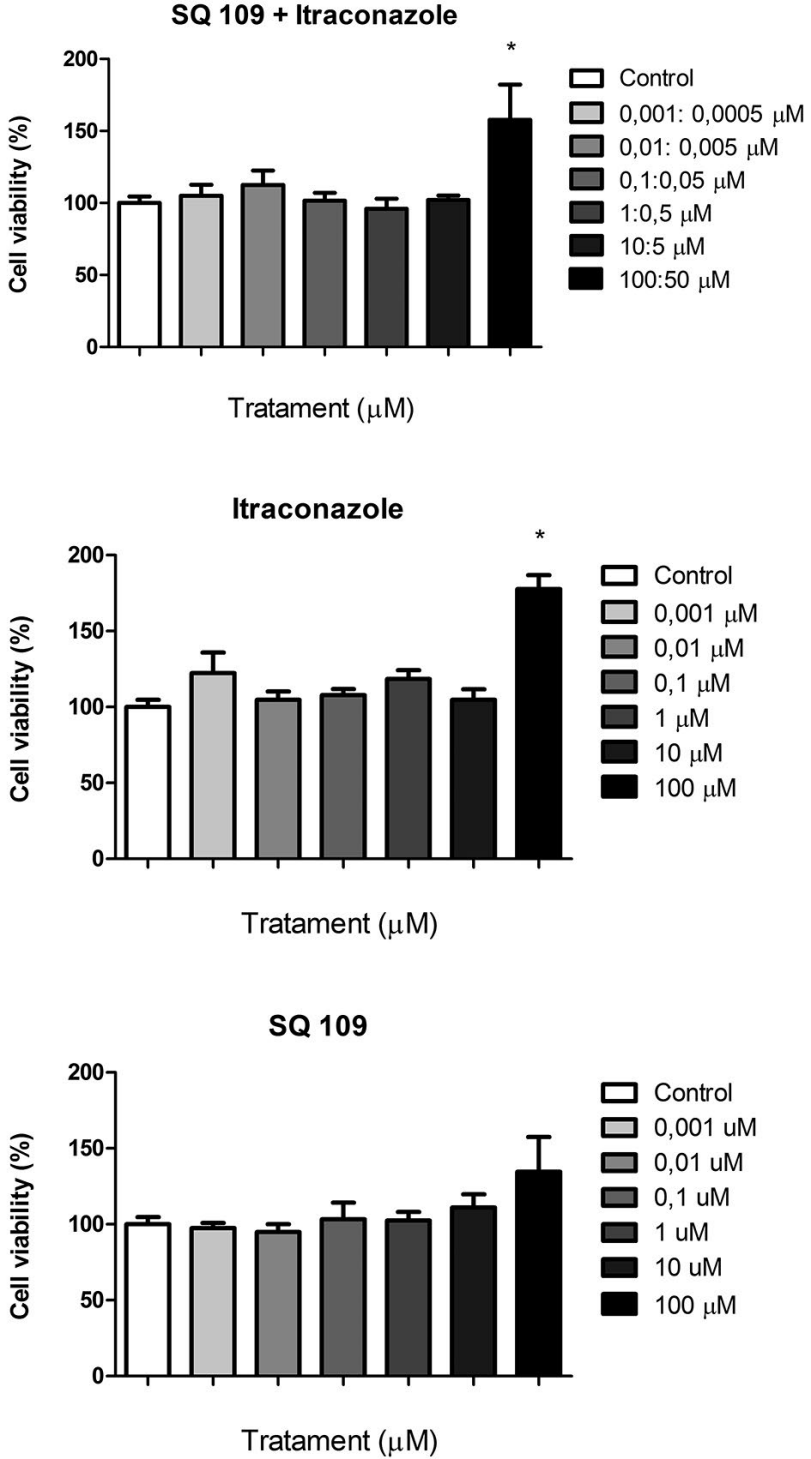
concentration-dependent cytotoxic effects (MTT assay) of compounds (SQ109 with itraconazole, respectively; itraconazole and SQ109) in a monkey kidney epithelial cells (VERO). Control wells received only the cell culture medium with or without (SQ109 with itraconazole; itraconazole and SQ109) addition of DMSO 1%. Results are expressed as % of total cell viability (spectrophotometric reads) in the culture medium after addition of a 5 mg/mL MTT solution (positive response). Histogram bar height is the mean ± standard error of the mean (SEM) of three independent experiments. P < 0.05, compared with the control group (0). MTT, 3-(4,5-dimethylthiazol-2-yl)-2,5-diphenyltetrazolium bromide.

*MMV687273 (SQ109) and MMV688415 are suitable for treatment in combination with itraconazole* - As presented by MMV, the CC_50_ MMV688415 in the HepG2 cell lineage is 38.4 µM. After the tests that showed a better response of SQ109, cytotoxicity assays were performed with VERO cells, where we observed that both NOEC and CC_50_ of SQ109 were higher than 100 µM (Fig. 2). The SI was determined with the compounds alone and in the presence of itraconazole (Table IV). The synergic interactions had a higher SI value for both compounds, being more selective for the pathogen. In addition, the combination of SQ109 and itraconazole was not toxic to VERO cells, even at high drug concentrations (Fig. 2).

**TABLE IV:** Selectivity index (SI) of the compounds presenting synergism with itraconazole against eight Herpotrichiellaceae species of clinical interest

Strains	MMV687273 (SQ109)		MMV688415	
	SI alone	SI after combination	SI alone	SI after combination
*Cladophialophora carrionii*	50	100	1.2	153.6
*Phialophora verrucosa*	25	6666.66	1.2	38.4
*Exophiala heteromorpha*	25	200	1.2	153.6
*Exophiala jeanselmei*	6.25	6.25	1.2	153.6
*Fonsecaea pedrosoi*	25	200	1.2	19.2
*Fonsecaea monophora*	25	400	1.2	153.6
*Fonsecaea nubica*	25	100	1.2	153.6
*Rhinocladiella similis*	12.50	25	1.2	153.6

Data was obtained with the following cell lines: VERO cells (SQ109), HepG2 cells (MMV688415); SI = CC_50_ (μM) / MIC (μM). The higher is the ratio obtained, the more selective is the compound against the fungal pathogen. MIC: minimum inhibitory concentration.

## DISCUSSION

One of the approaches to improve the treatment of fungal diseases is the combination of drugs presenting synergistic combination, which occurs when the combined effect of two drugs is greater than the sum of the individual activity of each drug.^47^ ^,^ ^48^ For example, cryptococcosis treatment with amphotericin B and 5FC is a successful therapy for most patients.^49^ In the CBM treatment, especially in relapse cases, the combination of two antifungal drugs is also common.^26^ ^,^ ^28^ ^,^ ^50^ ^,^ ^51^ Finding new synergistic combinations can improve the management of this infection.

When the Pathogen Box^®^ compound library was tested for the first time against some agents of CBM, the compounds MMV687273 (SQ109) and MMV688415 did not present relevant antifungal activity, with 37.41 and 13.61% of *F. pedrosoi* growth inhibition when tested alone, respectively.^37^ However, the screening of the substances in combination with sub-inhibitory concentrations of itraconazole, the most used antifungal drug to treat CBM and PHM, inhibited *F. pedrosoi* growth. The checkerboard method confirmed this synergic effect of both compounds against this species and, for MMV687273, also called SQ109, in more three Herpotrichiellaceae of clinical interest: *F. monophora* and *F. nubica*, which together with *F. pedrosoi* are the most common agents of CBM, and *E. dermatitidis*, which can cause PHM, CBM, and eumycotic mycetoma.^52^ This combination was proven to be non-toxic to mammalian cells and synergic for the majority of strains obtained from CBM cases, discarding species-specific effects. Moreover, the FICI obtained for the two strains without a synergic effect of this drug combination (FICI = 0.75) is described by some authors as additive, meaning that the combined effects of two drugs is equal to the sum of the effects of the two drugs acting independently.^53^

Synergistic interactions may also cause fungistatic drugs to switch to fungicides, providing a more effective treatment option.^54^ ^,^ ^55^ In the present study, we observed that itraconazole alone was fungistatic for all strains, while in combination with SQ109 was fungicidal for *F. pedrosoi* and *F. monophora* strains. Drug combination therapy can also decrease the toxicity of medications, by a reduction of doses required to achieve its biological effect. In fact, it was observed that both substances alone have a low SI when compared to their SI in combination with itraconazole. Reduction of itraconazole doses is interesting to the management of patients using other medications metabolised by the cytochrome P450 enzymes.^56^

SQ109 is a promising anti-*Mycobacterium tuberculosis* drug with a mechanism of action distinct from other antibiotics used in tuberculosis therapy. SQ109 inhibits cell wall synthesis and acts on multiple cellular pathways in a select group of microorganisms. Previous studies aiming to search for compounds with antifungal action found activity of the compound MMV687273 (SQ109) against *Candida albicans* biofilms^33^ and *Aspergillus fumigatus*.^57^ SQ109, in addition to exhibiting antibacterial activity against *M. tuberculosis*,^58^ is also active against *Trypanosoma cruzi* ^59^ and was recently proposed as a new drug for the treatment of Chagas disease,^59^ ^,^ ^60^ strongly suggesting its potential use for the treatment of different infectious diseases. This compound inhibits the trehalose monomycolate transporter MmpL3 in *M. tuberculosis*, which has homology with sphingolipid transporters found in fungi,^57^ suggesting a mechanism of action for this compound distinct to that presented by currently used antifungal drugs.

MMV688415 was originally classified as active against kinetoplastids. A previous study identified activity of this substance against *Leishmania aethiopica*.^61^ Another study showed that this class of substance, which is a trisubstituted pyridine derivative, has activity against *Sphaeropsis sapinea*, a pathogenic fungus of conifers.^62^ This data, together with the results of our study suggests that future works with trisubstituted pyridine derivatives may improve the options to treat fungal infections of plants and animals.

The present study indicates that, in combination with itraconazole, these substances, particularly SQ109, are promising candidates to treat *Fonsecaea* and *E. dermatitidis* infections, which account for most cases of CBM and PHM, respectively. They should be further studied for the development of new therapies in the treatment of these important mycoses.
